# Supplementary material for: Unplugging lateral fenestrations of NALCN reveals a hidden drug binding site within the pore region
Source: Proc Natl Acad Sci U S A. 2024 May 24;121(22):e2401591121. doi: 10.1073/pnas.2401591121 (PMC11145269; doi:10.1073/pnas.2401591121)
Supplement: Supplementary file 1 — Appendix 01 (PDF) [file pnas.2401591121.sapp.pdf]

PNAS

Supporting Information for
Unplugging lateral fenestrations of NALCN reveals a hidden drug binding site within the pore region

Katharina Schott[1], Samuel George Usher[1], Oscar Serra[2,3,4], Vincenzo Carnevale[2,3,4], Stephan Alexander Pless[1], Han Chow Chua[1,*,†]

[1]Department of Drug Design and Pharmacology, University of Copenhagen, Copenhagen, Denmark
[2]Department of Biology, Temple University, Philadelphia, PA, USA
[3]iGEM-Institute for Genomics and Evolutionary Medicine, Temple University, Philadelphia, PA, USA
[4]Institute of Computational Molecular Science, Temple University, Philadelphia, PA, USA

*Corresponding author. Email: chow.chua@sydney.edu.au
†Current address: Sydney Pharmacy School, Faculty of Medicine and Health and Charles Perkins Centre, The University of Sydney, NSW, Australia.

This PDF file includes:

Figures S1 to S7
Table S1

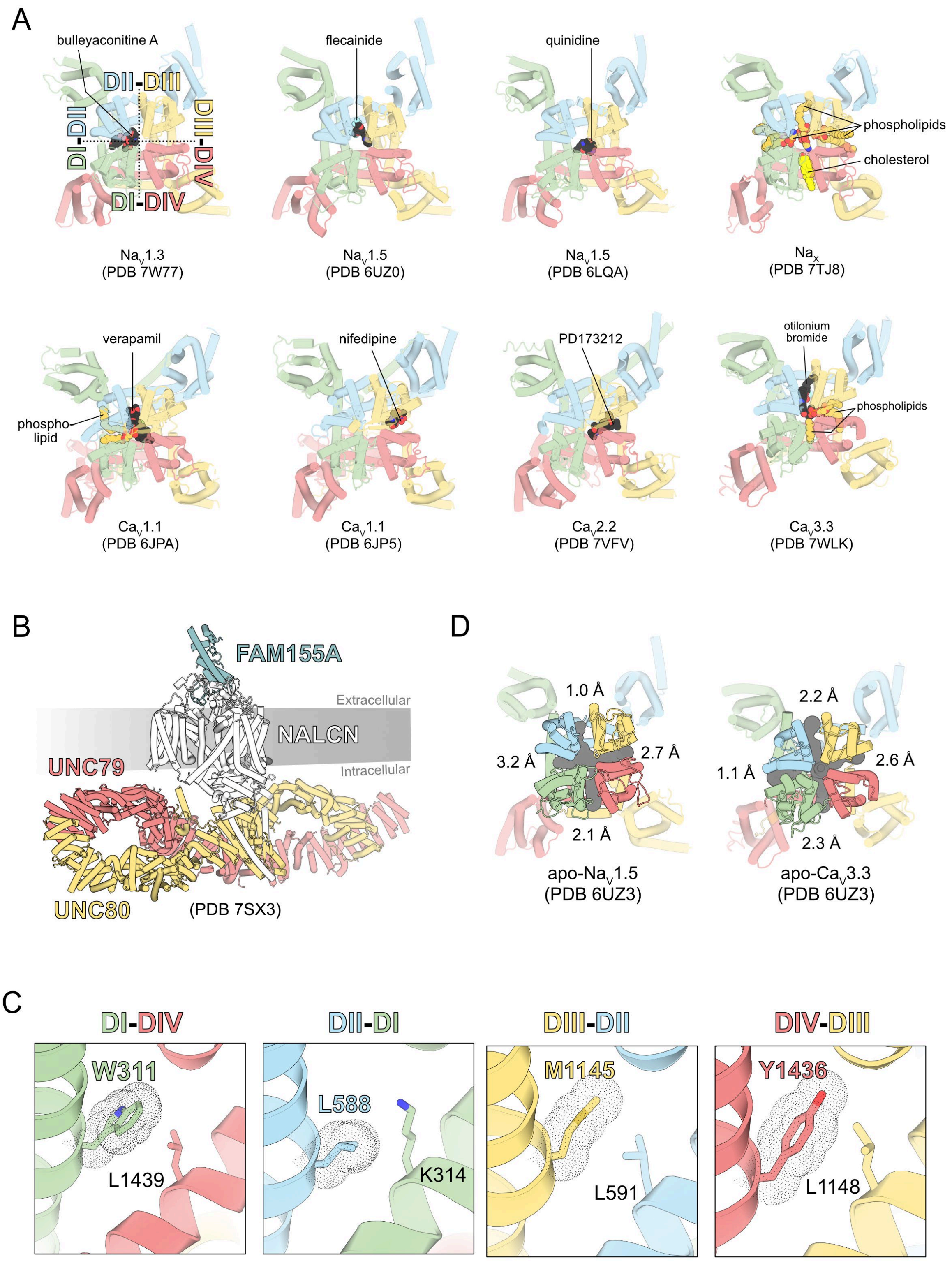

Fig. S1. (A) Top view of Na$_V$ and Ca$_V$ cryo-EM structures bound to blockers and phospholipids. (B) Side view of the NALCN channelosome structure. (C) Side views of NALCN WT showing the upward configuration of key bottleneck residues occluding the lateral fenestrations of NALCN. (D) Predicted lateral fenestrations of apo-Na$_V$1.5 (*top*) and apo-Ca$_V$3.3 (*bottom*) channels (top view). Bottleneck radii of individual predicted tunnels are indicated accordingly.

A

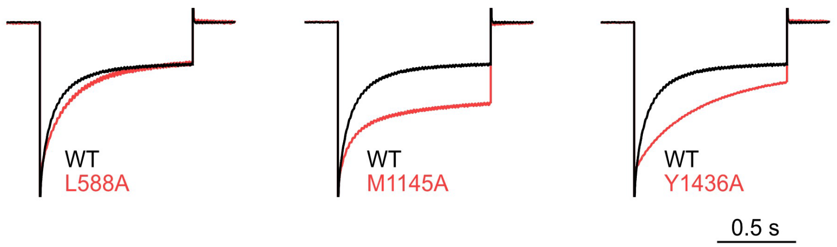

B

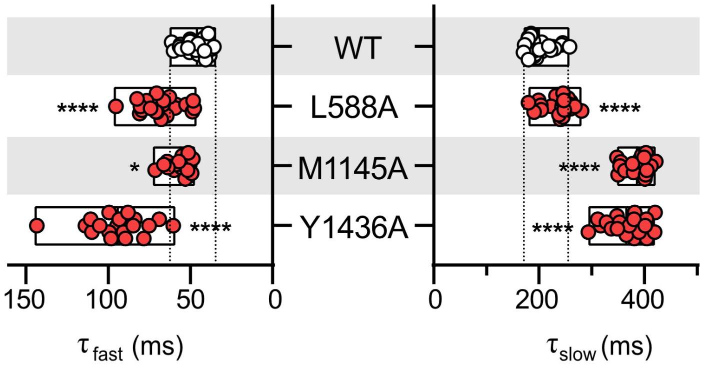

Fig. S2. (A) Superimposed normalised current traces of WT (black) and mutants (red) at -80 mV. (B) Slow and fast time constants of hyperpolarization-elicited currents (−80 mV) for WT and mutants. *p < 0.05; ****p < 0.0001; one-way ANOVA, Dunnett's test (against WT). See Supplementary Table 1 for descriptive statistics.

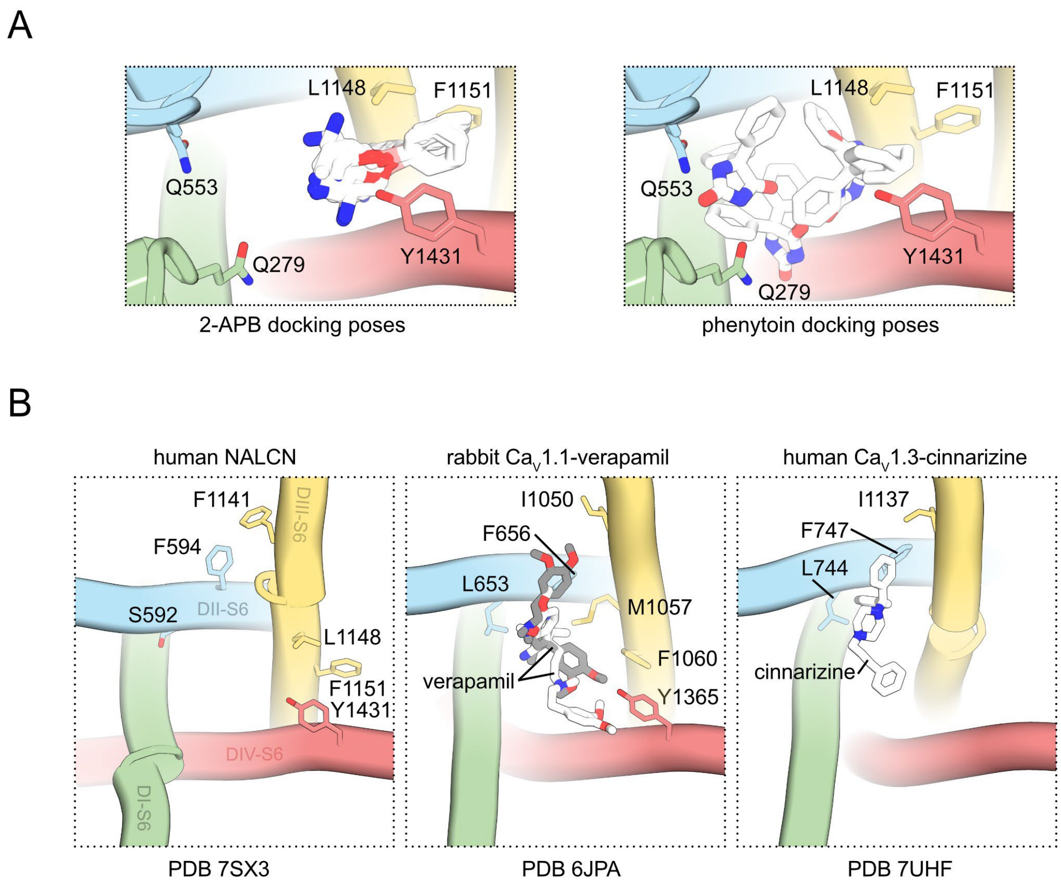

Fig. S3. (A) Top-ranked docked poses of 2-APB (left) and phenytoin (right) in the pore of NALCN AAAA. (B) *Left*, S6 segments of NALCN with residues that form the putative 2-APB binding sites within the pore region labelled. The corresponding residues in the rabbit Ca$_V$1.1-verapamil (*middle*) and human Ca$_V$1.3-cinnarizine (*right*) structures are highlighted.

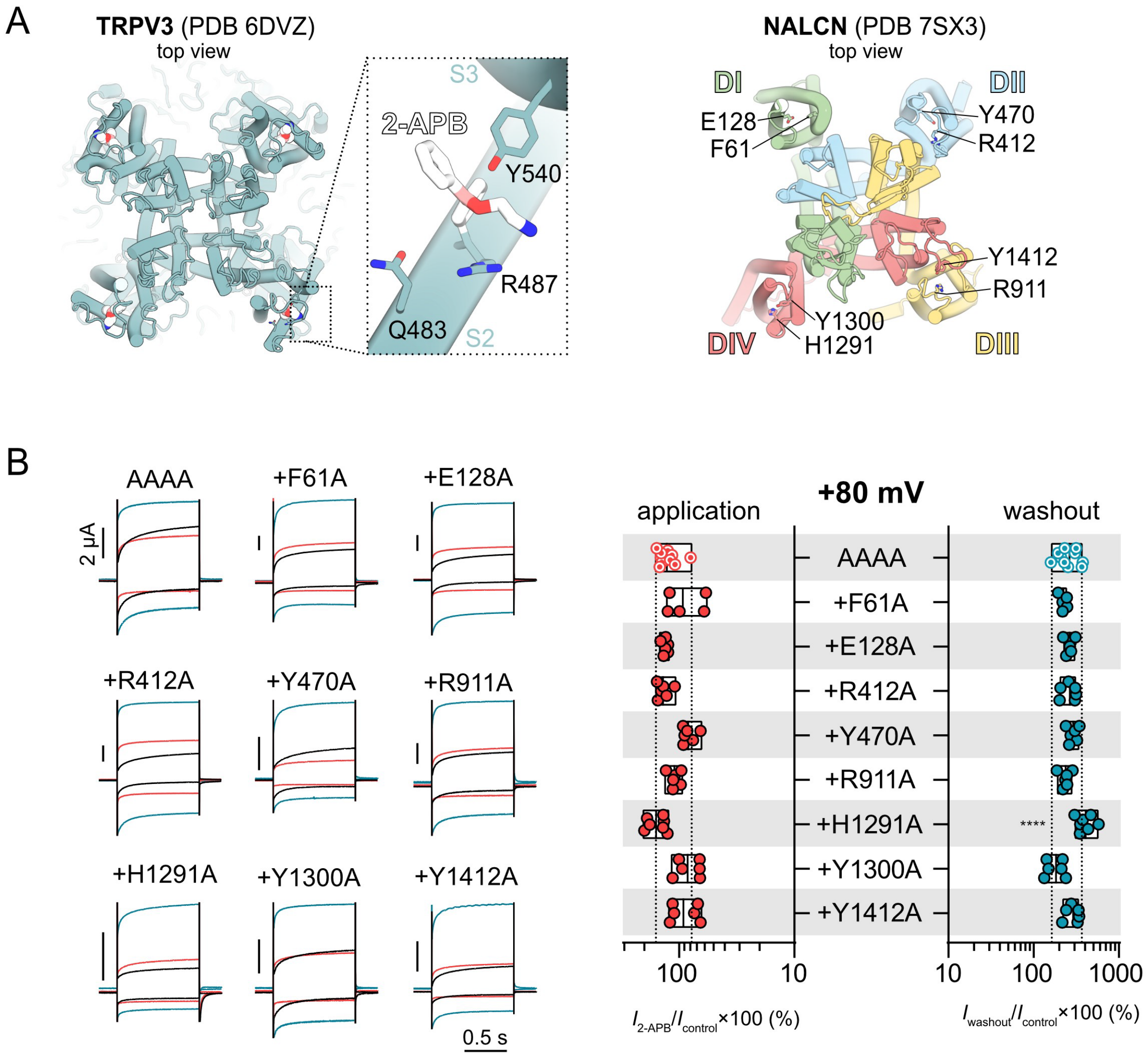

Fig. S4. (A) *Left*, top view of the TRPV3(Y564A)-2-APB structure (PDB 6DVZ), with expanded view of 2-APB molecule in a cavity formed by the extracellular portions of S1–S4 helices. 2-APB interacts with hydrophobic and hydrophilic residues including Q483, R487 and Y540. *Right*, top view of the NALCN structure (PDB 7SX3) with hydrophobic and hydrophilic residues around the extracellular side of individual VSDs labelled. (B) *Left*, representative current traces from *Xenopus laevis* oocytes expressing various mutant channels (on the background of NALCN AAAA) in response to application of 1 mM 2-APB. *Right*, the plot shows percentage of current left for NALCN AAAA and different mutants during 2-APB application and post 2-APB washout normalised against control current elicited at +80 mV. ****$p < 0.0001$; one-way ANOVA, Dunnett's test (against AAAA). See Supplementary Table 1 for descriptive statistics.

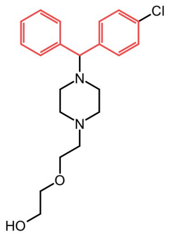

hydroxyzine

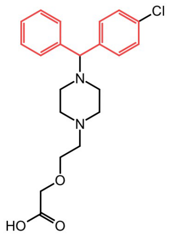

cetirizine

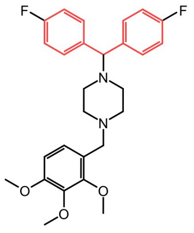

lomerizine

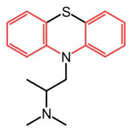

promethazine

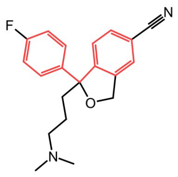

citalopram

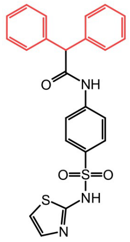

ICA-121431

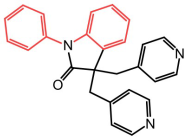

linopirdine

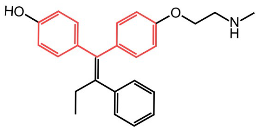

(E/Z)-endoxifen

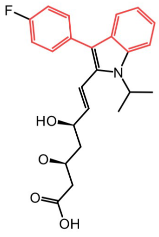

fluvastatin

Fig. S5. Chemical structures of compounds containing the diphenylmethane/amine motif (highlighted in red).

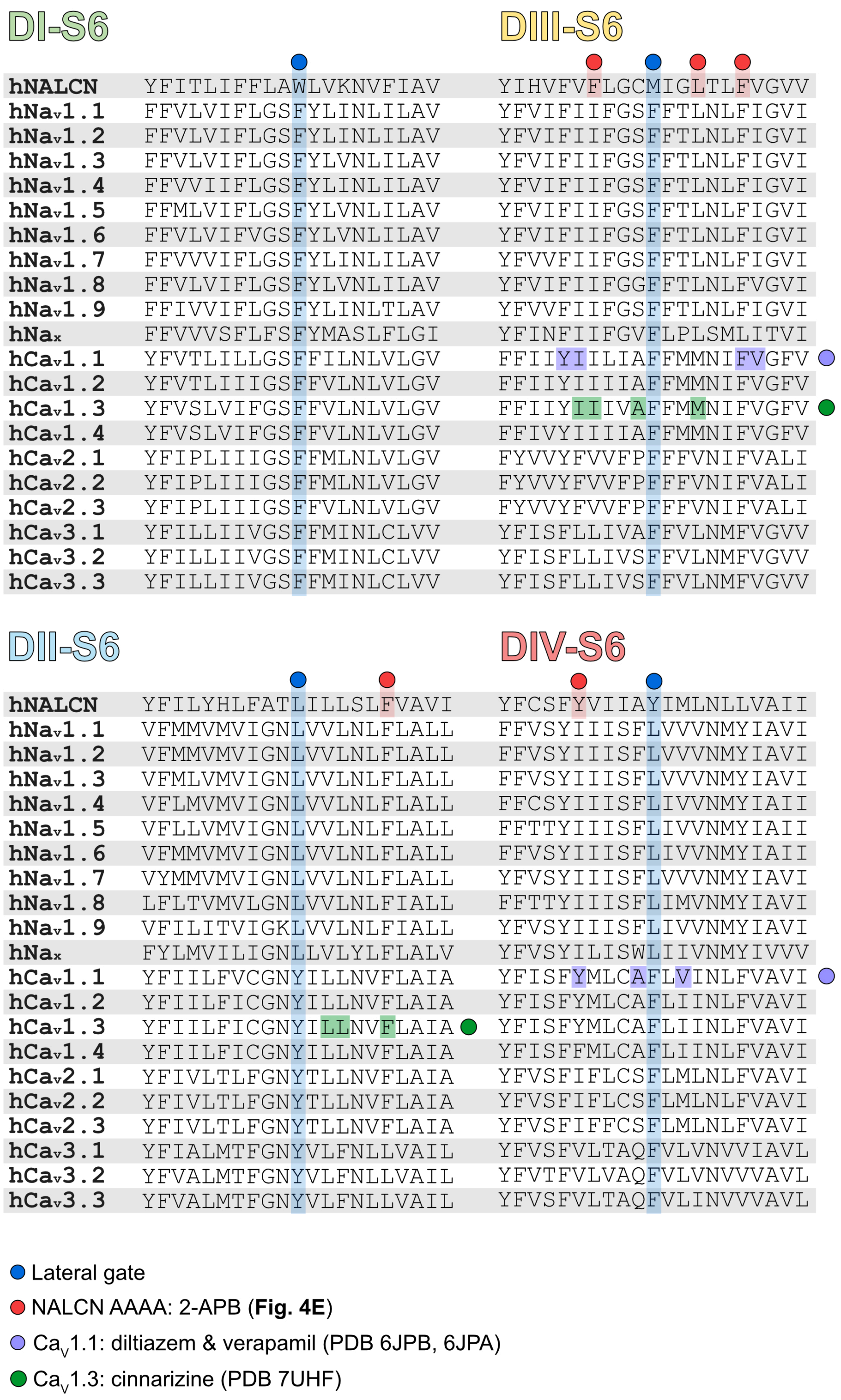

Fig. S6. Sequence alignment of the S6 segments of human NALCN, Na_V and Ca_V channels. The residues involved in forming the lateral fenestration gates, binding and function of various compounds are shaded in different colours.

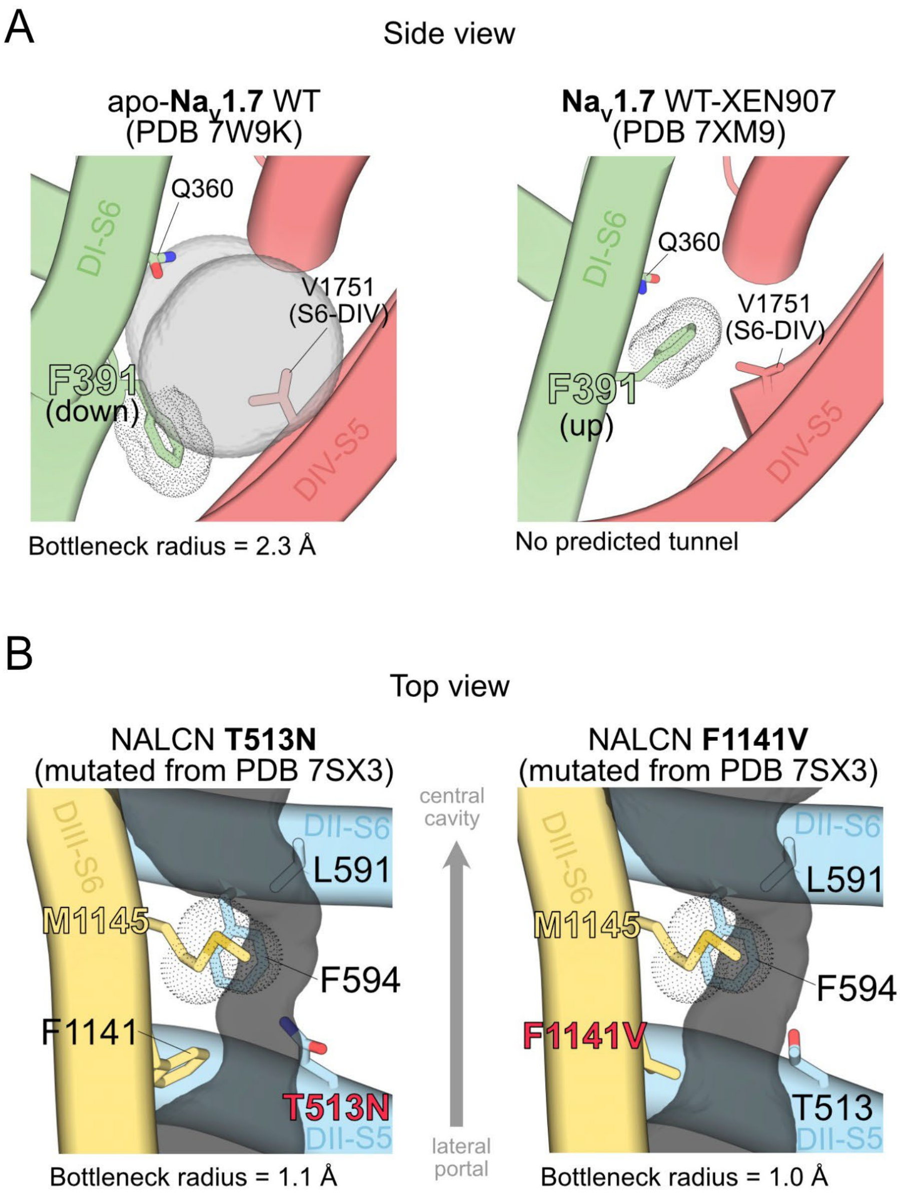

Fig. S7. (A) Side views of NaV1.7 showing the flexible conformations of a highly conserved phenylalanine residue (F391) in S6-DI. F391 appears to adopt a downward configuration in the structure of NaV1.7 WT in the absence of bound ligands (PDB 7W9K) and an upward configuration in the presence of the NaV1.7 blocker XEN907 (PDB 7XM9). (B) Top views of NALCN T513N (*left*) and F1141V (*right*) channels with predicted tunnels at DII-DIII interfaces shown as grey surfaces. Key bottleneck and fenestration-lining residues are labelled. The bottleneck radius values are indicated accordingly.

Table S1. Descriptive statistics for one-way ANOVA tests.

Figure 1C

Current amplitudes at +80 mV

	n	Mean (µA)	SD	95 % CI for mean		Min	Max
				Lower	Upper		
WT	73	4.363	0.8934	4.154	4.571	2.348	6.608
AAAA	75	3.675	0.6188	3.533	3.817	2.139	5.378
W311A	14	1.907	0.4844	1.627	2.187	1.213	3.104
L588A	18	3.888	0.9475	3.417	4.359	2.502	5.975
M1145A	17	4.134	0.9605	3.641	4.628	2.49	5.566
Y1436A	14	4.216	0.6582	3.836	4.596	3.201	5.392

Current amplitudes at -80 mV

	n	Mean (µA)	SD	95 % CI for mean		Min	Max
				Lower	Upper		
WT	73	-2.633	0.9528	-2.855	-2.41	-6.252	-1.486
AAAA	75	-1.935	0.6113	-2.075	-1.794	-4.916	-1.183
W311A	14	-0.5794	0.1462	-0.6638	-0.495	-0.9869	-0.4238
L588A	18	-2.394	0.6522	-2.718	-2.07	-4.297	-1.402
M1145A	17	-3.145	0.8808	-3.598	-2.692	-5.771	-2.185
Y1436A	14	-3.992	1.909	-5.095	-2.89	-7.224	-1.991

One-way ANOVA with Dunnett's test

Comparison	95 % confidence interval of difference	Adjusted *p* value	Summary
WT vs AAAA; +80 mV	0.3569 to 1.018	<0.0001	****
WT vs W311A; +80 mV	1.869 to 3.042	<0.0001	****
WT vs L588A; +80 mV	-0.05435 to 1.004	0.0988	ns
WT vs M1145A; +80 mV	-0.3134 to 0.7696	0.7820	ns
WT vs Y1436A; +80 mV	-0.4402 to 0.7331	0.9696	ns
WT vs AAAA; -80 mV	-1.073 to -0.3227	<0.0001	****
WT vs W311A; -80 mV	-2.719 to -1.388	<0.0001	****
WT vs L588A; -80 mV	-0.8392 to 0.3618	0.8193	ns
WT vs M1145A; -80 mV	-0.1024 to 1.127	0.1457	ns
WT vs Y1436A; -80 mV	0.6937 to 2.025	<0.0001	****

Figure 3A

% current left during phenytoin application at -100 mV (normalised against control current)

	n	Mean (%)	SD	95 % CI for mean		Min	Max
				Lower	Upper		
WT	12	101	9.089	95.24	106.8	87.98	121.4
AAAA	13	35.17	6.656	31.15	39.19	22.07	46.81
W311A	7	92.46	6.111	86.81	98.11	83.84	102.2
L588A	13	104.6	6.68	100.6	108.7	91.41	117.7
M1145A	11	69	9.157	62.85	75.15	52.42	79.25
Y1436A	12	103.4	3.254	101.4	105.5	99.01	110.3

One-way ANOVA with Dunnett's test

Comparison	95 % confidence interval of difference	Adjusted *p* value	Summary
WT vs AAAA	58.46 to 73.23	<0.0001	****
WT vs W311A	-0.2174 to 17.33	0.0584	ns
WT vs L588A	-11.01 to 3.761	0.5936	ns
WT vs M1145A	24.31 to 39.72	<0.0001	****
WT vs Y1436A	-9.948 to 5.119	0.8799	ns

Figure 3B

% current left after 2-APB washout at -100 mV (normalised against control current)

	n	Mean (%)	SD	95 % CI for mean		Min	Max
				Lower	Upper		
WT	17	123.7	11.67	117.7	129.7	107.7	147
AAAA	21	267.9	50.32	244.9	290.8	170.2	370.2
W311A	9	584	238.7	400.5	767.5	322.4	1066
W311F	8	138.4	19.82	121.8	154.9	113.7	165.3
W311A/L1439W	8	91.54	10.24	82.98	100.1	79.69	106.8
L588A	10	104.6	12.08	95.95	113.2	87.32	120.9
M1145A	7	117.1	19.9	98.66	135.5	83.46	138.6
Y1436A	9	109	8.468	102.5	115.5	95.55	122.6

One-way ANOVA with Dunnett's test

Comparison	95 % confidence interval of difference	Adjusted *p* value	Summary
WT vs AAAA	-215.9 to -72.44	<0.0001	****
WT vs W311A	-550.9 to -369.7	<0.0001	****
WT vs W311F	-108.9 to 79.57	0.9996	ns
WT vs W311A/L1439W	-62.07 to 126.4	0.9512	ns
WT vs L588A	-68.49 to 106.7	0.9963	ns
WT vs M1145A	-92.07 to 105.3	>0.9999	ns
WT vs Y1436A	-75.89 to 105.3	0.9994	ns

Figure 4E

% current left after 2-APB washout at +80 mV (normalised against control current)

	n	Mean (%)	SD	95 % CI for mean		Min	Max
				Lower	Upper		
AAAA	7	195.6	71.36	129.6	261.6	138.1	324.6
+Q279A	5	162.4	15.62	143.0	181.8	145.7	183.1
+S592A	5	123.7	18.27	101.0	146.4	105.8	148.0
+F594A	5	87.67	8.483	77.14	98.20	75.22	97.99
+F1141A	6	159.1	48.89	107.8	210.4	106.3	248.7
+L1148A	9	117.6	10.89	109.2	126.0	104.1	136.6
+F1151A	6	88.41	13.94	73.78	103.0	65.25	103.3
+Y1431A	6	63.33	12.09	50.64	76.02	49.41	81.80

One-way ANOVA with Dunnett's test; +80 mV

Comparison	95 % confidence interval of difference	Adjusted *p* value	Summary
AAAA vs +Q279A	-21.60 to 88.05	0.4246	ns
AAAA vs +S592A	17.07 to 126.7	0.0053	**
AAAA vs +F594A	53.10 to 162.8	<0.0001	****
AAAA vs +F1141A	-15.63 to 88.56	0.2789	ns
AAAA vs +L1148A	30.79 to 125.2	0.0003	***
AAAA vs +F1151A	55.09 to 159.3	<0.0001	****
AAAA vs +Y1431A	80.17 to 184.4	<0.0001	****

% current left after 2-APB washout at -100 mV (normalised against control current)

	n	Mean (%)	SD	95 % CI for mean		Min	Max
				Lower	Upper		
AAAA	7	152.6	37.18	118.3	187.0	110.4	223.5
+Q279A	5	125.7	11.00	112.0	139.3	110.0	139.7
+S592A	5	115.8	22.97	87.28	144.3	93.76	150.7
+F594A	5	33.93	7.553	24.55	43.30	21.94	41.38
+F1141A	6	101.5	56.18	42.52	160.4	64.14	212.9
+L1148A	9	63.08	6.944	57.75	68.42	52.91	71.06
+F1151A	6	41.39	10.63	30.23	52.54	28.15	54.86
+Y1431A	6	31.62	6.127	25.19	38.05	25.28	43.33

One-way ANOVA with Dunnett's test; -100 mV

Comparison	95 % confidence interval of difference	Adjusted *p* value	Summary
AAAA vs +Q279A	-15.03 to 68.99	0.3639	ns
AAAA vs +S592A	-5.167 to 78.85	0.1076	ns
AAAA vs +F594A	76.71 to 160.7	<0.0001	****
AAAA vs +F1141A	11.25 to 91.08	0.0067	**
AAAA vs +L1148A	53.40 to 125.7	<0.0001	****
AAAA vs +F1151A	71.34 to 151.2	<0.0001	****
AAAA vs +Y1431A	81.10 to 160.9	<0.0001	****

Figure 5B

% current left after compound application at +80 mV (normalised against control current of WT)

	n	Mean (%)	SD	95 % CI for mean		Min	Max
				Lower	Upper		
2-APB	17	79.22	23.29	67.25	91.20	50.92	133.6
DPH	9	39.71	4.347	36.37	43.05	34.73	48.09
Phenytoin	12	101.0	17.44	89.96	112.1	77.87	144.6
DPBA	7	33.07	4.040	29.33	36.80	28.56	40.46

% inhibition at +80 mV (normalised against control current of WT)

	n	Mean (%)	SD	95 % CI for mean		Min	Max
				Lower	Upper		
2-APB	17	20.78	23.29	8.802	32.75	-33.59	49.08
DPH	9	60.29	4.347	56.95	63.63	51.91	65.27
Phenytoin	12	-1.046	17.44	-12.13	10.04	-44.65	22.13
DPBA	7	66.93	4.040	63.20	70.67	59.54	71.44

One-way ANOVA with Dunnett's test (% inhibition); +80 mV

Comparison	95 % confidence interval of difference	Adjusted *p* value	Summary
2-APB vs DPH	-57.09 to -21.94	<0.0001	****
2-APB vs phenytoin	5.747 to 37.90	0.0051	**
2-APB vs DPBA	-65.30 to -27.01	<0.0001	****

% current left after compound application at -100 mV (normalised against control current of WT)

	n	Mean (%)	SD	95 % CI for mean		Min	Max
				Lower	Upper		
2-APB	17	31.50	7.647	27.57	35.43	16.83	46.06
DPH	9	184.9	19.80	169.7	200.1	154.7	212.8
Phenytoin	12	101.0	9.089	95.24	106.8	87.98	121.4
DPBA	7	21.00	6.058	15.40	26.61	14.05	32.73

% inhibition at -100 mV (normalised against control current of WT)

	n	Mean (%)	SD	95 % CI for mean		Min	Max
				Lower	Upper		
2-APB	17	68.50	7.647	64.57	72.43	53.94	83.17
DPH	9	-84.90	19.80	-100.1	-69.68	-112.8	-54.72
Phenytoin	12	-1.017	9.089	-6.792	4.757	-21.41	12.02
DPBA	7	79.00	6.058	73.39	86.40	67.27	85.95

One-way ANOVA with Dunnett's test (% inhibition); -100 mV

Comparison	95 % confidence interval of difference	Adjusted *p* value	Summary
2-APB vs DPH	142.0 to 164.8	<0.0001	****
2-APB vs phenytoin	59.05 to 79.98	<0.0001	****
2-APB vs DPBA	-22.96 to 1.965	0.1179	ns

Figure S2B

Fast time constants of inward currents at -80 mV

	n	Mean (ms)	SD	95 % CI for mean		Min	Max
				Lower	Upper		
WT	27	45.88	7.188	43.03	48.72	35.69	61.17
L588A	24	68.46	11.96	63.41	73.51	47.61	95.12
M1145A	18	55.87	6.688	52.55	59.20	48.41	71.39
Y1436A	19	93.90	18.52	84.97	102.8	60.32	143.1

One-way ANOVA with Dunnett's test (fast time constants)

Comparison	95 % confidence interval of difference	Adjusted *p* value	Summary
WT vs L588A	-30.50 to -14.67	<0.0001	****
WT vs M1145A	-18.58 to -1.409	0.0179	*
WT vs Y1436A	-56.47 to -39.57	<0.0001	****

Slow time constants of inward currents at -80 mV

	n	Mean (ms)	SD	95 % CI for mean		Min	Max
				Lower	Upper		
WT	27	170.4	23.86	190.8	209.7	170.4	258.2
L588A	24	180.2	26.67	224.3	246.9	180.2	281.0
M1145A	18	348.2	22.91	374.5	397.3	348.2	421.7
Y1436A	19	293.4	37.23	348.1	384.0	293.4	419.9

One-way ANOVA with Dunnett's test (slow time constants)

Comparison	95 % confidence interval of difference	Adjusted *p* value	Summary
WT vs L588A	-54.10 to -16.56	<0.0001	****
WT vs M1145A	-206.0 to -165.3	<0.0001	****
WT vs Y1436A	-185.8 to -145.7	<0.0001	****

Figure S4B

% current left after 2-APB application at +80 mV (normalised against control current)

	n	Mean (%)	SD	95 % CI for mean		Min	Max
				Lower	Upper		
AAAA	10	127.4	22.95	111	143.8	79.27	155.5
+F61A	5	92.55	31.79	53.08	132.0	58.5	124.7
+E128A	6	131.5	7.691	123.5	139.6	123.8	144.1
+R412A	6	137.0	17.19	119	155.1	108.5	154.6
+Y470A	6	82.84	10.74	71.57	94.11	64.86	92.00
+R911A	6	109.1	12.45	96	122.1	95.12	129.1
+H1291A	7	157.2	30.28	129.2	185.2	125.2	198.5
+Y1300A	6	84.15	20.89	62.22	106.1	65.77	113.2
+Y1412A	6	91.65	24.97	65.44	117.9	64.95	119.5

One-way ANOVA with Dunnett's test; +80 mV (2-APB application)

Comparison	95 % confidence interval of difference	Adjusted *p* value	Summary
AAAA vs +F61A	1.881 to 67.76	0.0333	*
AAAA vs +E128A	-35.22 to 26.89	0.9998	ns
AAAA vs +R412A	-40.73 to 21.38	0.9579	ns
AAAA vs +Y470A	13.47 to 75.58	0.0016	**
AAAA vs +R911A	-12.75 to 49.35	0.4975	ns
AAAA vs + H1291A	-59.42 to -0.1497	0.0483	*
AAAA vs +Y1300A	12.17 to 74.27	0.0023	**
AAAA vs +Y1412A	4.671 to 66.78	0.0166	**

We only consider a comparison significantly different if the adjusted *p* value is <0.0001.

% current left after 2-APB washout at +80 mV (normalised against control current)

	n	Mean (%)	SD	95 % CI for mean		Min	Max
				Lower	Upper		
AAAA	10	264.2	73.62	211.5	316.9	160.0	375.7
+F61A	5	224.7	22.62	196.6	252.8	192.9	247.8
+E128A	6	264.3	30.29	232.6	296.1	222.9	310.5
+R412A	6	265.6	52.02	211.0	320.2	201.2	314.7
+Y470A	6	290.2	39.55	248.7	331.7	239.3	343.7
+R911A	6	236.5	32.64	202.2	270.7	187.1	285.7
+H1291A	7	409.6	93.01	323.6	495.6	303.5	579.5
+Y1300A	6	182.3	45.84	134.2	230.4	133.2	239.2
+Y1412A	6	290.1	53.59	233.8	346.3	216.3	342.9

One-way ANOVA with Dunnett's test; +80 mV (2-APB washout)

Comparison	95 % confidence interval of difference	Adjusted *p* value	Summary
AAAA vs +F61A	-47.59 to 126.6	0.7683	ns
AAAA vs +E128A	-82.27 to 81.99	>0.9999	ns
AAAA vs +R412A	-83.54 to 80.72	>0.9999	ns
AAAA vs +Y470A	-108.1 to 56.15	0.9543	ns
AAAA vs +R911A	-54.40 to 109.9	0.9360	Ns
AAAA vs + H1291A	-223.8 to -67.04	<0.0001	****
AAAA vs +Y1300A	-0.2493 to 164.0	0.0511	ns
AAAA vs +Y1412A	-108.0 to 56.26	0.9553	ns
